# The pseudorabies virus UL13 protein kinase triggers phosphorylation of the RNA demethylase FTO, which is associated with FTO-dependent suppression of interferon-stimulated gene expression

**DOI:** 10.1128/jvi.02019-24

**Published:** 2025-01-10

**Authors:** Ruth Verhamme, Robert J. J. Jansens, Jianheng Liu, Fien Van Raemdonck, Cliff Van Waesberghe, Luke Nicholson, Samie R. Jaffrey, Herman W. Favoreel

**Affiliations:** 1 Department of Translational Physiology, Infectiology and Public Health, Faculty of Veterinary Medicine, Ghent University 366759 , Merelbeke, Belgium; 2 Department of Pharmacology, Weill Medical College, Cornell University 271509 , New York, New York, USA; The University of Arizona, Tucson, Arizona, USA

**Keywords:** pseudorabies virus, PRV, alphaherpesvirus, UL13, m6Am, epitranscriptome, FTO, interferon, interferon-stimulated genes, PICF1, m6A

## Abstract

Alpha-ketoglutarate-dependent dioxygenase, also known as fat mass and obesity-associated protein (FTO), is an RNA demethylase that mediates the demethylation of N^6^,2-O-dimethyladenosine (m6Am) and N^6^-methyladenosine (m6A). Both m6Am and m6A are prevalent modifications in mRNA and affect different aspects of transcript biology, including splicing, nuclear export, translation efficiency, and degradation. The role of FTO during (herpes) virus infection remains largely unexplored. In this study, we show that the UL13 protein kinase of the alphaherpesvirus pseudorabies virus (PRV) triggers phosphorylation of FTO. In primary epithelial cells, depletion of FTO leads to increased expression of antiviral interferon-stimulated genes (ISGs) and UL13 triggers FTO-dependent suppression of ISG expression. Although PRV infection suppresses m6Am levels in host small nuclear RNA, this is independent of UL13. The current data highlight FTO as an important regulator of antiviral ISG expression and suggest that UL13-mediated phosphorylation of FTO may serve as a previously unrecognized viral strategy to suppress the antiviral interferon response.

**IMPORTANCE:**

RNA modification pathways play important roles in diverse cellular processes and virus life cycles. Although previous studies have demonstrated that alphaherpesviruses can substantially influence cellular RNA modifications, such as m6A, the impact on the m6Am epitranscriptome machinery remains largely unexplored. The present work reports that the UL13 protein kinase of pseudorabies virus (PRV), an alphaherpesvirus, mediates phosphorylation of the m6Am/m6A eraser FTO and that this correlates with a UL13- and FTO-dependent suppression of antiviral interferon-stimulated gene (ISG) expression. Furthermore, PRV infection leads to a pronounced reduction in m6Am levels in host snRNA and also induces phosphorylation of the m6Am writer PCIF1. These data highlight FTO as an important regulator of ISG expression and reveal that viral manipulation of FTO, such as UL13-induced phosphorylation of FTO, may serve as a previously unrecognized interferon evasion strategy.

## INTRODUCTION

The diverse landscape of post- and co-transcriptional modifications in RNA is called the epitranscriptome. Among these, N^6^-methyladenosine (m6A) and N^6^,2-O-dimethyladenosine (m6Am) stand out as prevalent modifications found within mRNA and other RNA molecules, including small nuclear RNA (snRNA) (1, 2). Despite their chemical resemblance, the distribution patterns of m6A and m6Am are remarkably different. While m6A predominates at the onset of the last exon of mRNAs (3, 4), m6Am is mainly situated at the first adenosine adjacent to the 5′ cap structure (5). The m6A modification has been found in various RNA species, including mRNA, ribosomal RNA, small nuclear RNA (snRNA), and microRNA. The presence of m6Am has been detected only in mRNA and snRNA so far (2, 6).

Both types of modifications are enzymatically installed by methyltransferases. M6A is typically deposited by a multi-subunit methyltransferase complex called the m6A writer complex (7). In contrast, m6Am is incorporated in an m7G cap-dependent manner by the methyltransferase PCIF1 (8–11). An internal m6Am modification in U2 snRNA is deposited by the methyltransferase METTL4, a paralog of METTL3 (12).

The first enzyme that was reported to demethylate m6A modifications on mRNA was characterized as the fat mass and obesity-associated protein (FTO), also known as alpha-ketoglutarate-dependent dioxygenase. This finding indicated that RNA modifications are reversible (13). Later research showed that m6Am may serve as the main physiological substrate of FTO, with a nearly 100-fold higher catalytic activity of FTO toward m6Am compared to m6A (5, 14). Meanwhile, the ketoglutarate-dependent dioxygenase alkB homolog 5 (ALKBH5) was identified as an m6A demethylase and does not act on m6Am (15).

In recent years, mounting evidence has highlighted the significance of the epitranscriptome and the epitranscriptome machinery in virus infections (16). Modulation of m6A writers or erasers through knockdown or overexpression affects the replication of viruses across diverse virus families (17, 18). The impact of RNA methylation on virus replication appears multifaceted. On the one hand, methylation of viral mRNA can exert direct effects on the viral transcriptome that often result in enhanced virus replication compared to viruses expressing unmethylated transcripts (19). On the other hand, there is a growing body of research that underscores the role of RNA methylation in regulating the antiviral immune response, particularly the type I IFN response (20). Despite considerable progress in the field of viral epitranscriptomics, information on how viruses affect the epitranscriptome machinery and the exact mechanisms through which RNA methylation affects virus replication remain poorly understood.

We and others have recently demonstrated that alphaherpesvirus infection has a very substantial impact on the host m6A epitranscriptome due to preferential degradation of m6A-containing host transcripts and a viral inhibition of the m6A writer complex (21–24). However, little is known about the potential impact of alphaherpesvirus infection on m6A(m) eraser proteins and the role of these proteins in virus infection. In this study, we unveil that infection of cells with the porcine alphaherpesvirus PRV leads to phosphorylation of the m6A(m) demethylase FTO. We show that FTO phosphorylation critically depends on the expression of the UL13 protein, a viral serine/threonine protein kinase. The PRV UL13 kinase is not only necessary but also sufficient for FTO phosphorylation, as the sole expression of kinase-active UL13 is sufficient to trigger FTO phosphorylation. FTO depletion in primary epithelial cells leads to a strong upregulation of ISG expression, which correlates with a significantly reduced viral protein expression. In addition, primary epithelial cells infected with UL13null PRV display increased ISG expression levels compared to cells infected with wild-type (WT) PRV, and FTO knockdown increases ISG expression in WT PRV-infected cells to the level observed in UL13null PRV-infected cells. These data, therefore, suggest that phosphorylation of FTO serves as a viral mechanism to suppress the antiviral type I IFN response. Overall, our findings reveal a previously uncharacterized impact of alphaherpesvirus infection on FTO and the antiviral IFN response.

## MATERIALS AND METHODS

### Cells and viruses

Swine testicle (ST) cells were cultured in MEM (Gibco) supplemented with 10% fetal calf serum (FCS), 1 mM sodium pyruvate, 10^5^ U/L penicillin, 100 mg/L streptomycin, and 50 mg/L gentamycin. HEK293T cells were cultured in DMEM (Gibco) supplemented with 10% FCS, 10^5^ U/L penicillin, 100 mg/L streptomycin, and 50 mg/L gentamicin. Both cell lines were obtained from the American Type Culture Collection and were tested to be free of mycoplasma contamination via de LookOut Mycoplasma PCR detection kit (Sigma-Aldrich).

Primary porcine kidney (PPK) cells were isolated as described previously (25) and were cultured in MEM (Gibco) supplemented with 10% FCS, 10^5^ U/L penicillin, and 50 mg/L gentamycin. Cells were kept at 37°C in a humidified atmosphere at 5% CO_2_.

An FTO knockout (KO) ST cell line was generated using the CRISPR-Cas9 system from IDT. A CRISPR RNA (crRNA) against the third exon of porcine FTO was custom designed using the design tool from IDT (design ID: CD.Cas9.FWJR4400.BS). The custom crRNA and the trans-activating RNA (tracrRNA) (1075927, IDT) were annealed by heating to 95°C for 5 minutes. The CRISPR-Cas9 RNP was formed by mixing the annealed guide RNA with the recombinant Cas9 protein (1081058, IDT) and incubating it for 5 minutes at room temperature. The CRISPR-Cas9 RNP was transfected using lipofectamine RNAiMAX (Thermo Fisher). Six days after transfection, cells were collected, and single cells were isolated. Single cells were expanded and checked for FTO expression by Western blotting. DNA from the FTO-negative cell line was isolated, and FTO was amplified and sequenced to identify the mutation in the gene. A deletion of a single nucleotide in the third exon caused a frame shift leading to the absence of FTO in this cell line.

WT NIA3 PRV strain and an isogenic US3null and UL13null mutant (M137) were previously described and were kindly donated by the ID-DLO (The Netherlands) (26–28). For immunofluorescence assays, monomeric red fluorescent protein-labeled VP26-labeled Becker WT PRV (GS847) and UL13null PRV (GS1018) strains were used, which were described previously (29, 30). Confluent cells were infected at a multiplicity of infection (MOI) of 10 and lysed at 16 hours post-infection (hpi) unless otherwise mentioned.

### Cell treatments

The 26S proteasome inhibitor MG132 was purchased from Merck, and treatment was performed for 4 hours at a concentration of 10 µM, after which cells were lysed for Western blotting. In Fig. 5E, cells were treated 2 hpi at a concentration of 10 µM, and treatment remained until 8 hpi when cells were lysed for RNA extraction. A plasmid encoding recombinant porcine IFN-alpha was kindly provided by Simon Yongming (Kansas State University, USA). This plasmid was transfected into HEK293T cells, and the supernatant was collected 48 hours post-transfection (hpt). The amount of IFN-alpha secreted in the supernatant was measured by enzyme-linked immunosorbent assay as described before (31). IFN-alpha treatment was performed by treating the cells with 300 ng/mL IFN-alpha at 2 hpi.

### Transfections and plasmids

The pTrip plasmid encoding enhanced green fluorescent protein (GFP) was a kind gift from B. Verhasselt (Ghent University, Ghent, Belgium). Plasmids encoding WT or kinase defective (KD) UL13 were previously in-house generated (23). Cells were transfected with 2,250 ng of plasmid DNA for each well of a 6-well plate using JetPrime (Polyplus) according to the manufacturer’s instructions. Transfected cells were lysed and analyzed at 48 hpt.

### FTO siRNA-mediated knockdown

FTO was knocked down using the DsiRNA system from IDT. siRNAs targeting porcine FTO were designed using the design tool from IDT (design ID: CD.Ri.120323.13.5). siRNAs were transfected using lipofectamine RNAiMAX according to the manufacturer’s instructions (ThermoFisher). In brief, 2.5 µL of a 10 µM stock of siRNA and 7.5 µL of lipofectamine RNAiMAX were used for one well of a 6-well plate. All experiments were performed using scrambled negative control DsiRNA as a negative control (51-01-19-09, IDT). At 24 and 48 hpt for PPK cells and ST cells, respectively, knockdown cells were infected and processed further.

### Western blotting

Cells were lysed at 48 hpt or 16 hpi, unless mentioned otherwise, in RIPA buffer (Abcam) with cOmplete mini EDTA-free protease inhibitor cocktail (Roche) and PhosStop (Roche). Cell lysates were separated on a 10% polyacrylamide gel, followed by blotting on the PVDF membrane (Amersham). Blots were blocked in 5% nonfat milk diluted in 0.1% Tween-20 in PBS for 1 hour at room temperature. Primary antibodies were incubated overnight at 4°C. Following three consecutive 5-minute washes in PBS-T, the membranes were incubated with the secondary antibody for 1 hour at room temperature. Following three more 5-minute washes, the blots were detected using Pierce enhanced chemiluminescence (ECL) substrate (Thermo Scientific), ECL Plus substrate (GE Healthcare), or SuperSignal West Femto maximum sensitivity substrate (Thermo Scientific) on a ChemiDoc MP imaging device (Bio-Rad).

Phos-tag gels were purchased from Fujifilm and analyzed identically, except using a lysis buffer lacking EDTA and performing EDTA treatment of the gels before blotting to remove zinc ions for optimal transfer efficiency.

Treatment of lambda protein phosphatase (New England Biolabs) was performed according to the manufacturer’s instructions.

Western blot assays were performed using primary antibodies against alpha-tubulin (Abcam ab40742, 1/1,000), phosphorylated FTO (Abcam, ab92821, 1/500), FTO (Abcam, ab124892, 1/1,000), FTO (Proteintech, 27226-1-AP, 1/1,000), ALKBH5 (Abcam, ab195377, 1/1,000), PRV US3 (1/100) (32), PRV UL13 (1/1,000) (33), PRV gE (1/100) (34), STAT3 (Cell Signaling Technology, 4904, 1/2,000), phosphorylated STAT3 (Cell Signaling Technology, 9145, 1/2,000), and PCIF1 (Proteintech, 16082-1-AP, 1/1,000).

### RNA isolation and reverse transcriptase quantitative PCR (RT-qPCR)

RNA isolations were performed using the RNeasy Mini Kit (Qiagen) according to the manufacturer’s procedure. Purified RNA was treated with RNase-free DNase I (New England Biolabs) at 37°C for 10 minutes to remove contaminating DNA. To stop DNase I activity, EDTA (Invitrogen) was added at a final concentration of 5 mM, and samples were incubated at 75°C for 10 minutes. Reverse transcription was performed with 500 ng RNA using an iScript cDNA synthesis kit (Bio-Rad) according to the manufacturer’s instructions. Cycling was performed in a StepOnePlus real-time PCR system (Applied Biosystems, Thermo Fisher Scientific) with SYBR green master mix (Applied Biosystems, Thermo Fisher Scientific). The relative expression of each gene was analyzed by the double delta threshold cycle method and normalized to the level of expression of the 28S rRNA gene, which has been validated as a reference gene as previously described (35). Primers used for the different genes are listed in Table 1.

**TABLE 1:** Primers used in RT-qPCR

Target	Forward primer (5′−3′)	Reverse primer (5′−3′)	Reference
28S	GGGCCGAAACGATCTCAACC	GCCGGGCTTCTTACCCAT	36
2′5′OAS	GAGCTGCAGCGAGACTTCCT	TGCTTGACAAGGCGGATGA	37
ISG15	AGCACAGTCCTGTTGATGGTG	CAGAACTGGTCAGCTTGCACG	37
ISG54	GCCCTAAGGACCCAGAAGTCA	CGAGGAGGTGGCCAGTTATC	37
UL13	AGCCACCTGGACGTCAAGG	CCATGAGGCTAAAGTCCCCG	36
gC	TCGTGAGCAGCATGATCGT	GTCGCCATGATGACCAGC	36
FTO	TGACTGCCATCCTAGCCGTG	GGCAGAGTTCGGGCAATTCG	This paper

### Virus titrations

Confluent cell monolayers were infected at an MOI of 10. Virus inoculum was washed away at 2 hpi, and the cells were washed twice with PBS. The cells were treated with sodium citrate buffer, pH 3.0 (40 mM sodium citrate, 10 mM KCl, and 135 mM NaCl), for 2 minutes at room temperature to remove all remaining infectious virus from the input (38). Following two more washing steps with PBS, fresh medium was added.

After the time of infection that was mentioned, the infectious virus in the supernatants was titrated by 1/10 serial dilution assays on ST cells, and four experimental repeats were performed. The characteristic PRV-derived cytopathic effect served as a readout. Titers are expressed as the log10 of the TCID_50_/mL.

### Immunofluorescence staining

Cells were fixed using 3% paraformaldehyde for 10 minutes after which they were permeabilized using methanol for 10 minutes. Primary antibodies were incubated overnight at 4°C. Following three 5-minute washing steps with PBS, cells were incubated with secondary antibody for 1 hour at 37°C at a 1/200 dilution. After three more washing steps with PBS, cells were mounted using glycerine-DABCO. Stained samples were imaged using a Dmi8 Thunder inverted microscope (Leica). Images were processed and analyzed using FIJI. Immunofluorescence assays were performed using a primary antibody against FTO (Abcam, ab124892, 1/300), and specificity was validated using FTO KO ST cells.

### CROWN-seq library preparation

The recently developed CROWN-seq technique was used as a quantitative 5′ m6Am mapping method (39). Total RNA was isolated using the Quick-RNA Miniprep kit (Zymo Research) according to the manufacturer’s instructions. Approximately 30 µg of total RNA input was used for the CROWN-seq libraries. The RNA input was converted by sodium nitrite as described in the GLORI protocol (40). After conversion, the m7G capped 5′ end RNA fragments were isolated based on a modified ReCappable-seq workflow (41). In brief, the converted m7G capped RNAs were first recapped by 3′-Desthiobiotin-GTP. Then, the recapped RNA fragments were enriched on streptavidin beads. Different from the original ReCappable-seq protocol, we performed on-beads 3′ adapter ligation, where a barcoded 3′ DNA adapter (Table 2) was ligated to the enriched RNA fragments. We used four different barcodes to label different samples. After adapter ligation, two to four samples in different barcodes were pooled. This pooling strategy can increase the yield of the library. The pooled RNA fragments were then treated by 5′ deadenylase (NEB #M0331S) and RecJf (NEB #M0264S), which can remove the exceeded 3′ adapter. After a second round of streptavidin enrichment, the RNA fragments were eluted into water. To ligate the 5′ adapter, we used RppH (NEB #M0356S) to release the 3′-Desthiobiotin caps, which results in monophosphate 5′ ends ready for ligation. We then ligated a 5′ adapter (Table 2), which contains a unique molecular identifier (UMI) to the RNA fragments. After adapter ligation, reverse transcription primer (Table 2) was annealed to the 3′ adapter (75°C for 5 minutes, 37°C for 15 minutes, and then 25°C for 15 minutes). Reverse transcription was performed with SuperScript III (Thermo Fisher #18080093). After reverse transcription, cDNA was amplified with Phusion PCR master mix (NEB #M0531S) and NEBNext multiplex oligos (NEB #E7780S). Amplified libraries were size selected by SPRIselect beads (Beckman #B23317). Size-selected libraries were then sequenced by NovaSeqX in PE100 mode.

**TABLE 2:** Sequences needed for CROWN-seq analysis

Sequence type	Sequence
3′ adapter sequences	/rApp/ANNNNNNNNTCGCAGATCGGAAGAGCACACGTCTGAACTCCAGTCACAAAAAAAAAAAAAAACCCCCCCCCCAAAAAAAAAAAAAAA/3AmMO/
	/rApp/ANNNNNNNNGCACAGATCGGAAGAGCACACGTCTGAACTCCAGTCACAAAAAAAAAAAAAAACCCCCCCCCCAAAAAAAAAAAAAAA/3AmMO/
	/rApp/ANNNNNNNNTGATAGATCGGAAGAGCACACGTCTGAACTCCAGTCACAAAAAAAAAAAAAAACCCCCCCCCCAAAAAAAAAAAAAAA/3AmMO/
	/rApp/ANNNNNNNNGTCCAGATCGGAAGAGCACACGTCTGAACTCCAGTCACAAAAAAAAAAAAAAACCCCCCCCCCAAAAAAAAAAAAAAA/3AmMO/
5′ adapter sequence	rCrCrUrArCrArCrGrArCrGrCrUrCrUrUrCrCrGrArUrCrUrArUrNrN
Reverse transcription primer sequence	GACGTGTGCTCTTCCGATCT

### Bioinformatics analysis of m6Am mapping and quantification

To obtain reads for individual libraries from the pooled sequencing results, we performed two steps of processing by Cutadapt3. First, we removed the low-quality reads by parameters: cutadapt -a ANNNNNNNNNNNNAGATCGGAAGAGCACACGTC -A NNTAAGATCGGAAGAGCGTCGTG --max-n 0 -m 32 -q 20 -e 0.25. Second, we extracted different libraries by pattern recognition: cutadapt -j 8 -g [sample #1]=GCGA -g [sample #2]=GTGC -g [sample #3]=ATCA -g [sample #4]=GGAC. The extracted reads were stored in different FASTQ files for further analysis.

To analyze individual libraries, we first removed the AT adapter sequences at the beginning of the read (40). Then, the UMI sequences in both read1 and read2 were extracted by UMI-tools (42) (umitools extract --bc-pattern2 NNNNNNNNN -p NN). To map the reads to a reference genome, we first A-to-G and T-to-C converted the reference genome of pig (Ensembl Sscrofa11.1 r110) and PRV (GCA_000843825.1) *in silico*. We then built HISAT2 indexes on these converted reference genomes (43). The reads were mapped to the converted reference genome by the CROWN-seq alignment pipeline (https://github.com/jhfoxliu/CROWN-Seq). Notably, only unique alignments were used in analyses. After alignment, we removed PCR duplications by UMI-tools. We then calculated the coverages of the 5′ end mapped to the reference genome, which represents the positions of transcription-start nucleotides. For A-started transcripts, the m6Am level was calculated as the number of A (m6Am) reads versus the sum of A reads and G (Am) reads. To annotate snRNA genes, we used snRNA annotations in Ensembl Sscrofa11.1 r110.

### Statistical analysis

For the statistical analysis of the results of Fig. 6, normality was checked by the Shapiro-Wilk test, and a two-sided Mann-Whitney *U* test was used. For the statistical analysis of the results of Fig. 5, normality was checked by the Shapiro-Wilk test, and a one-tailed Mann-Whitney *U* test was used. *P*-values < 0.05 were considered to be significant. Visualization and statistical analysis of the data were performed in Jupyter Notebook using Seaborn for Fig. 6 and in GraphPad Prism for Fig. 5.

## RESULTS

### PRV infection triggers FTO phosphorylation

**Fig 1:**
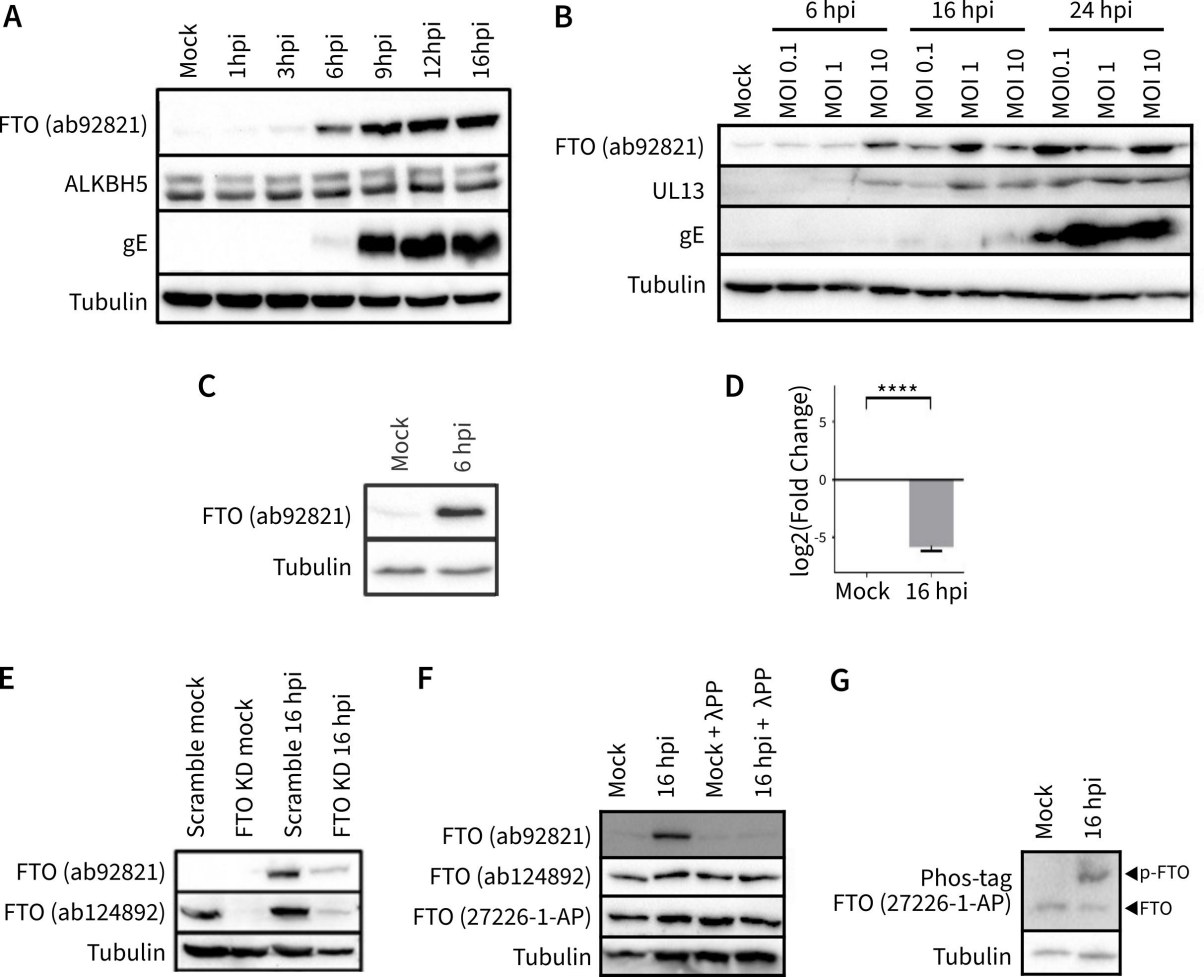
PRV infection triggers phosphorylation of the m6A(m) eraser FTO. (**A**) Western blot analysis of FTO and ALKBH5 upon infection of ST cells with PRV at different hpi. Tubulin and viral gE signals serve as loading and infection controls, respectively. Western blot was generated with the same sample set and at the same time as that in Fig. 2A of reference 23. Infection (gE) and loading (tubulin) control signals are, therefore, the same in both figure panels. (**B**) Western blot analysis of FTO upon infection of ST cells with PRV at different MOIs and different hours post-infection. Tubulin and viral UL13/gE signals serve as loading and infection controls, respectively. (**C**) Western blot analysis of PRV-infected HEK293T cells at 6 hpi. (**D**) FTO mRNA expression in ST cells infected with PRV for 16 hours, relative to mock-infected cells. ****P < 0.0001. (**E**) Western blot analysis of ST cells treated with 10 µM of the proteasome inhibitor MG132 for 4 hours or infected with PRV for 6 hours. (**F**) Western blot analysis of FTO signal in mock- or PRV-infected ST cells at 16 hpi upon siRNA-mediated knockdown (KD) of FTO (or treatment with scrambled siRNA). λ-phosphatase (λPP) treatment of mock- or PRV-infected samples collected at 16 hpi. (**G**) Phos-tag analysis of FTO in ST cells that were mock infected or infected with PRV and analyzed at 16 hpi.

First, we assessed whether PRV infection affects the expression of the m6A(m) erasers ALKBH5 and/or FTO. Western blot analysis of porcine ST cells analyzed at various time points after PRV infection revealed a pronounced increase in FTO signal, starting around 6 hpi, while ALKBH5 protein band intensity remained unchanged (Fig. 1A). The increase in FTO signal was also evident at lower MOIs (Fig. 1B). Upon infection at an MOI of 0.1 and 1, PRV-induced phosphorylation of FTO could be detected at 16 and 24 hpi, while infection at an MOI of 10 phosphorylation triggers detectable FTO phosphorylation already at 6 hpi. This effect was cell type or host independent as the increase in FTO signal was also observed in PRV-infected human HEK293T cells (Fig. 1C).

To assess whether the PRV-induced increase in FTO protein band intensity on Western blot was associated with increased levels of FTO mRNA, reverse transcriptase-quantitative PCR (RT-qPCR) analysis was performed. Surprisingly, FTO transcripts were reduced upon PRV infection (Fig. 1D). While this FTO transcript downregulation aligns with earlier described viral mechanisms to impair host transcription, including host shutoff function (44) and broad inhibition of cellular gene expression during PRV infection (45), it contrasts the increased FTO protein signal observed via Western blotting.

FTO protein levels can be regulated by proteasomal degradation following ubiquitination (46). To explore whether the increase in FTO protein signal in PRV-infected cells could correspond to an inhibition of proteasomal degradation of FTO, mock-infected cells were treated with the proteasome inhibitor MG132. FTO protein band intensity of mock-infected cells that were treated for 4 hours with the proteasome inhibitor MG132 still was not readily detectable and much weaker than the signal observed in PRV-infected cells (data not shown), arguing against reduced proteasomal FTO degradation as an explanation for the increased FTO protein band intensity in PRV-infected cells.

An alternative explanation for the observed increase in FTO signal could be that the anti-FTO antibody that was used (ab92821, Abcam) preferentially recognizes a specific subset of (post-translationally modified) FTO. To assess this, we used an additional anti-FTO antibody (ab124892, Abcam) that targets another region of the FTO protein. Upon siRNA-mediated FTO knockdown, Western blotting signal was strongly downregulated for both antibodies, indicating their specificity for FTO (data not shown). Peculiarly, the alternative anti-FTO antibody did not show an increased signal in Western blot upon PRV infection (Fig. 1E). This suggests that the ab92821 antibody preferentially recognizes a specific form of FTO, possibly post-translationally modified FTO.

Since phosphorylation represents one of the most abundant types of protein post-translational modification, we assessed whether the increase in FTO signal upon PRV infection was due to the recognition of a phosphorylated form of FTO by antibody ab92821.

To this end, samples from mock- and PRV-infected cells were treated with phosphatase. Treatment with phosphatase completely reversed the increase in FTO signal detected by antibody ab92821 in PRV-infected cells, whereas no change in signal was observed when using the ab124892 antibody or yet another anti-FTO antibody (27226-1-AP, Proteintech) (Fig. 1F). These data suggest that the ab92821 antibody preferentially recognizes a phosphorylated version of FTO that is more abundantly present upon PRV infection. To confirm that PRV infection triggers FTO phosphorylation, Phos-tag gels, which increase differences in the migration speed of phosphorylated proteins, were used. These assays revealed the presence of a distinct FTO band with a prominent mobility shift upon infection of WT PRV, thereby confirming that PRV infection triggers phosphorylation of FTO (Fig. 1G), while no evidence was found for phosphorylation of the m6A eraser ALKBH5 (data not shown).

In summary, our findings indicate that PRV infection triggers phosphorylation of FTO.

### Expression of the PRV UL13 protein kinase is necessary and sufficient to trigger FTO phosphorylation

**Fig 2:**
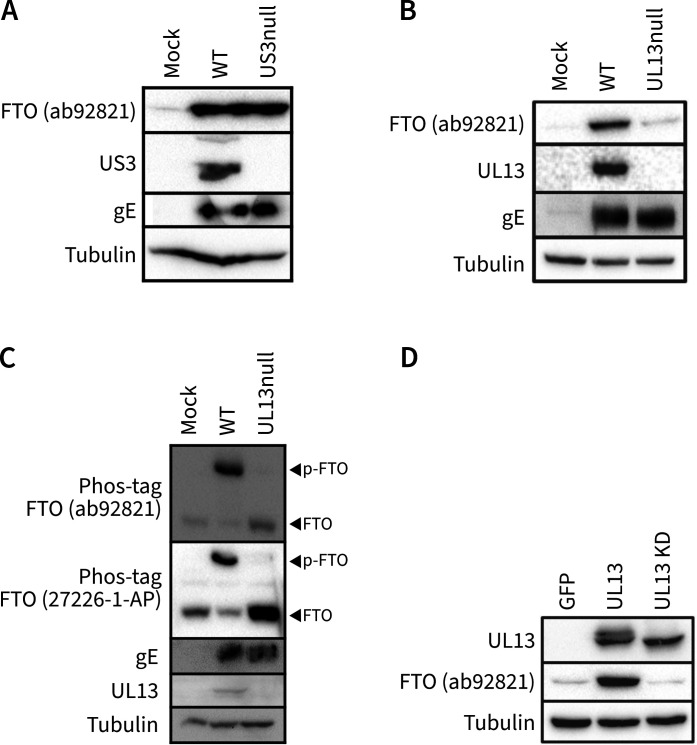
Expression of the viral UL13 protein kinase is essential and sufficient to trigger FTO phosphorylation. (**A**) Western blot analysis of ST cells infected with WT or isogenic US3null PRV at 16 hpi. (**B**) Western blot analysis of ST cells infected with WT or isogenic UL13null PRV at 16 hpi. (**C**) Phos-tag analysis of FTO in ST cells that were mock-infected or infected with WT or isogenic UL13null mutant PRV strains at 16 hpi. (**D**) Western blot analysis of ST cells transfected with GFP, kinase-intact or kinase defective (KD) UL13 at 48 hpt.

The PRV genome encodes two viral protein kinases, US3 and UL13. Infection of ST cells with a mutant virus that does not express US3 yielded similar levels of FTO phosphorylation compared to WT infection (Fig. 2A). However, infection with a virus mutated in the UL13 gene failed to induce the accumulation of phosphorylated FTO (Fig. 2B), indicating that the viral UL13 kinase is required for FTO phosphorylation.

To confirm this, Phos-tag gels were used to check the FTO migration pattern in ST cells infected with WT or UL13null PRV. Phos-tag assays using the 27226-1-AP anti-FTO antibody revealed that most of the FTO protein signal in WT PRV-infected cells was derived from phosphorylated FTO, while only the non-phosphorylated FTO protein signal was observed in UL13null PRV-infected cells (Fig. 2C). In addition, a similar Phos-tag assay using the ab92821 anti-FTO antibody confirmed that this antibody preferentially recognizes phosphorylated FTO, as it yielded a strong signal from the phosphorylated FTO protein band in WT PRV-infected cells, but only a weak signal from the non-phosphorylated band in UL13null PRV-infected or mock-infected cells (Fig. 2C).

Next, we aimed to assess whether the expression of the UL13 protein kinase alone, in the absence of virus infection, is sufficient to trigger FTO phosphorylation. For this purpose, transfection assays were performed using plasmids encoding WT kinase-active UL13 or kinase defective (KD) UL13. The latter has an aspartate-to-alanine mutation in the catalytic domain of the kinase (D194A) (23, 29). Both plasmids resulted in abundant UL13 protein expression, although the UL13 protein band of the kinase-inactive UL13 variant lacks a slower migrating band on Western blot analysis. This is likely attributable to the absence of UL13 autophosphorylation, a characteristic shared by all known UL13 homologs (47). Expression of kinase-intact UL13 triggered phosphorylation of FTO, while expression of KD UL13 did not (Fig. 2D).

In conclusion, the expression of UL13 is necessary and sufficient to trigger the phosphorylation of FTO, and FTO phosphorylation depends on the kinase activity of UL13.

### PRV UL13-induced FTO phosphorylation does not affect subcellular FTO localization

Immunofluorescence studies have consistently shown that FTO is mainly located within the nucleus of the cell (13, 48–50). In line with this, FTO harbors a functional nuclear localization signal in its N-terminal domain (51, 52). To assess whether PRV-induced FTO phosphorylation affects its subcellular localization, immunofluorescence imaging was performed on mock-infected ST cells as well as ST cells infected with WT or UL13null PRV.

**Fig 3:**
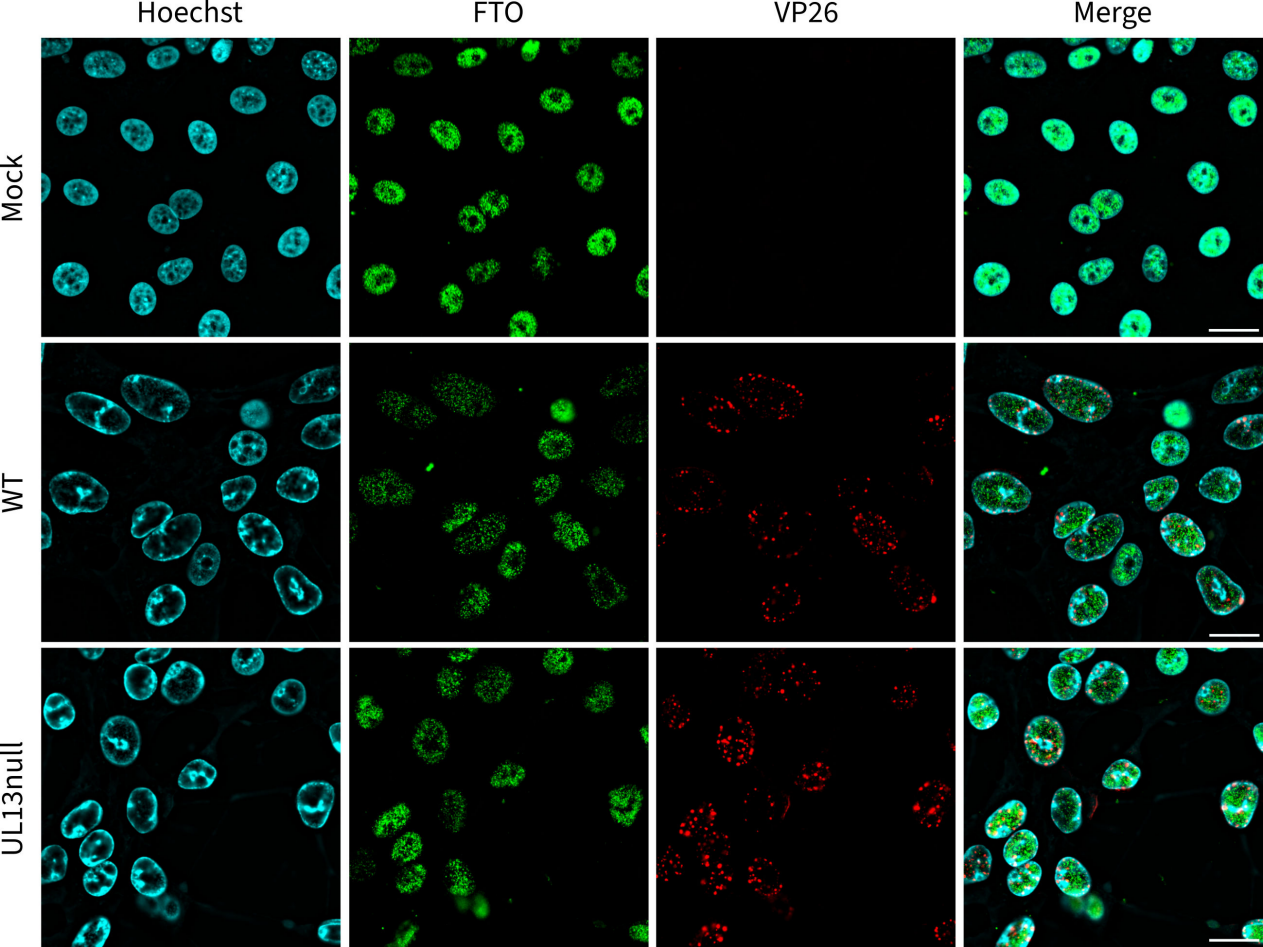
UL13 expression does not affect subcellular FTO localization. Immunofluorescence imaging of FTO (green) in mock-infected ST cells and ST cells infected with WT or isogenic UL13null PRV at 8 hours post-infection. Cell nuclei were counterstained with Hoechst (blue), and cells were infected with monomeric red fluorescent protein-labeled VP26 (red) PRV strains. Scale bar, 20 µm.

This revealed that FTO primarily resides in the nuclei of mock-infected ST cells (Fig. 3), consistent with earlier research (13, 48, 50). Upon PRV infection, FTO remained predominantly nuclear, although its distribution appeared punctate compared to the homogenous distribution observed in mock-infected cells. FTO localization was similar in cells infected with WT or UL13null PRV, suggesting that PRV-induced alteration in sub-nuclear FTO localization is UL13 independent (Fig. 3). In line with this, immunofluorescence imaging of ST cells transfected with either kinase-intact or KD UL13 did not reveal substantial differences in FTO localization (data not shown).

Taken together, these data indicate that, although PRV infection appears to alter sub-nuclear location of FTO, UL13-induced phosphorylation does not affect subcellular localization of FTO.

### FTO does not contribute to PRV replication in ST cells

**Fig 4:**
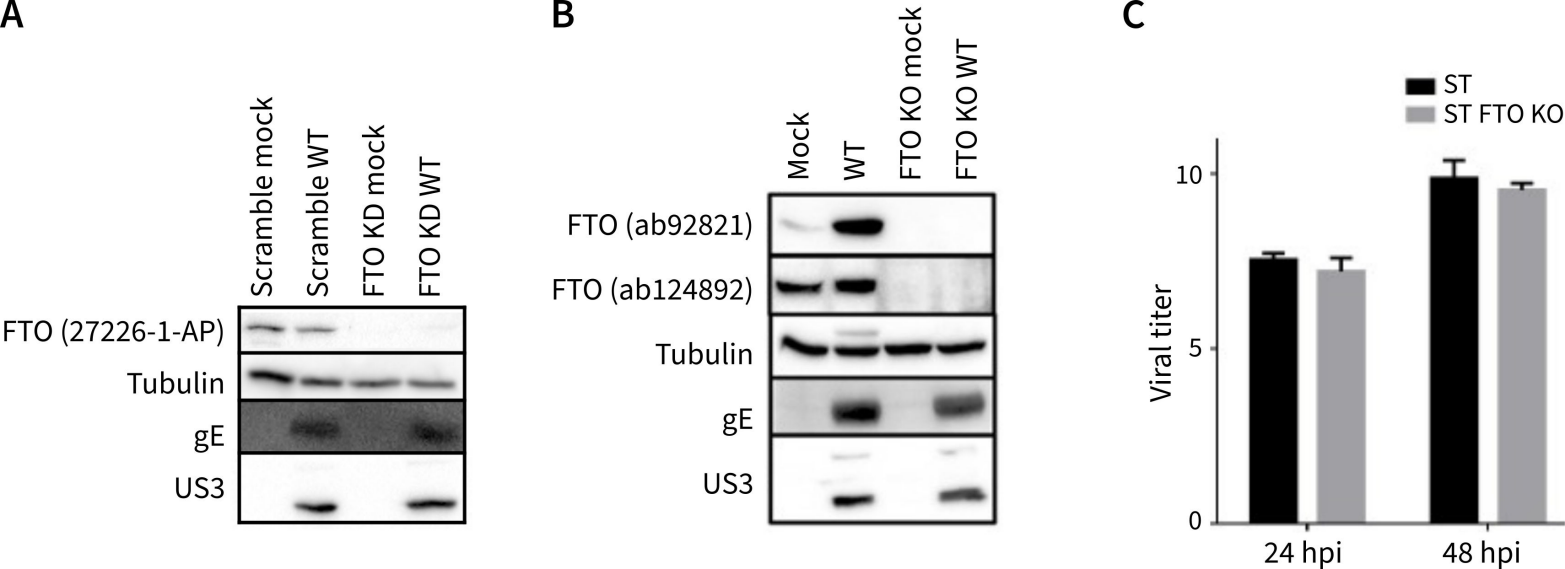
FTO is not required for PRV replication in cell culture. (**A**) Western blot assay of early (US3) and late (gE) viral protein production in mock or wild-type PRV-infected ST cells at 16 hours post-infection upon siRNA-mediated KD of FTO or treatment with scrambled siRNA. (**B**) Western blot assay of early (US3) and late (gE) viral protein production in mock- or WT PRV-infected parental ST cells or ST cells KO for FTO at 16 hpi. (**C**) Infectious virus titers at 24 and 48 hpi in PRV-infected parental ST cells and ST FTO KO cells. Titers are expressed as the log10 of the TCID50/mL.

To assess whether FTO contributes to the PRV replication cycle in cell culture, we performed an siRNA-mediated knockdown of FTO in ST cells, followed by PRV infection. Western blot analysis confirmed successful knockdown of FTO. However, FTO knockdown did not substantially affect viral protein expression upon infection (Fig. 4A). The limited effect on viral protein production suggests that FTO is not essential in the virus replication cycle in cell culture, although it cannot be excluded that residual activity of remaining FTO protein following knockdown may mask a potential role of FTO in PRV replication.

To address the latter, an FTO knockout (FTO KO) ST cell line was generated via CRISPR-Cas9 technology. Characterization of this cell line revealed a frameshift mutation in the third exon of FTO, resulting in complete absence of detectable FTO protein. Consistent with our observations in FTO knockdown cells, PRV infection of FTO KO ST cells resulted in only a minor reduction in viral protein production (Fig. 4B). In addition, titration of progeny virus produced in PRV-infected cells showed that PRV infection of FTO KO ST cells resulted in only slightly reduced virus titers compared to normal ST cells, but no statistically significant differences were observed (Fig. 4C).

Overall, these data indicate that FTO does not play a substantial role in PRV replication efficiency in the ST cell line.

### FTO knockdown leads to increased expression of interferon-stimulated genes and suppressed PRV protein production, and UL13 triggers FTO-dependent suppression of ISG expression in primary porcine epithelial cells

Given that ST cells, like many immortalized cell lines (53), are defective in type I IFN production (24), the potential impact of FTO and/or modulation of FTO by PRV on the production of type I IFN and ISGs cannot be readily evaluated in this cell type.

**Fig 5:**
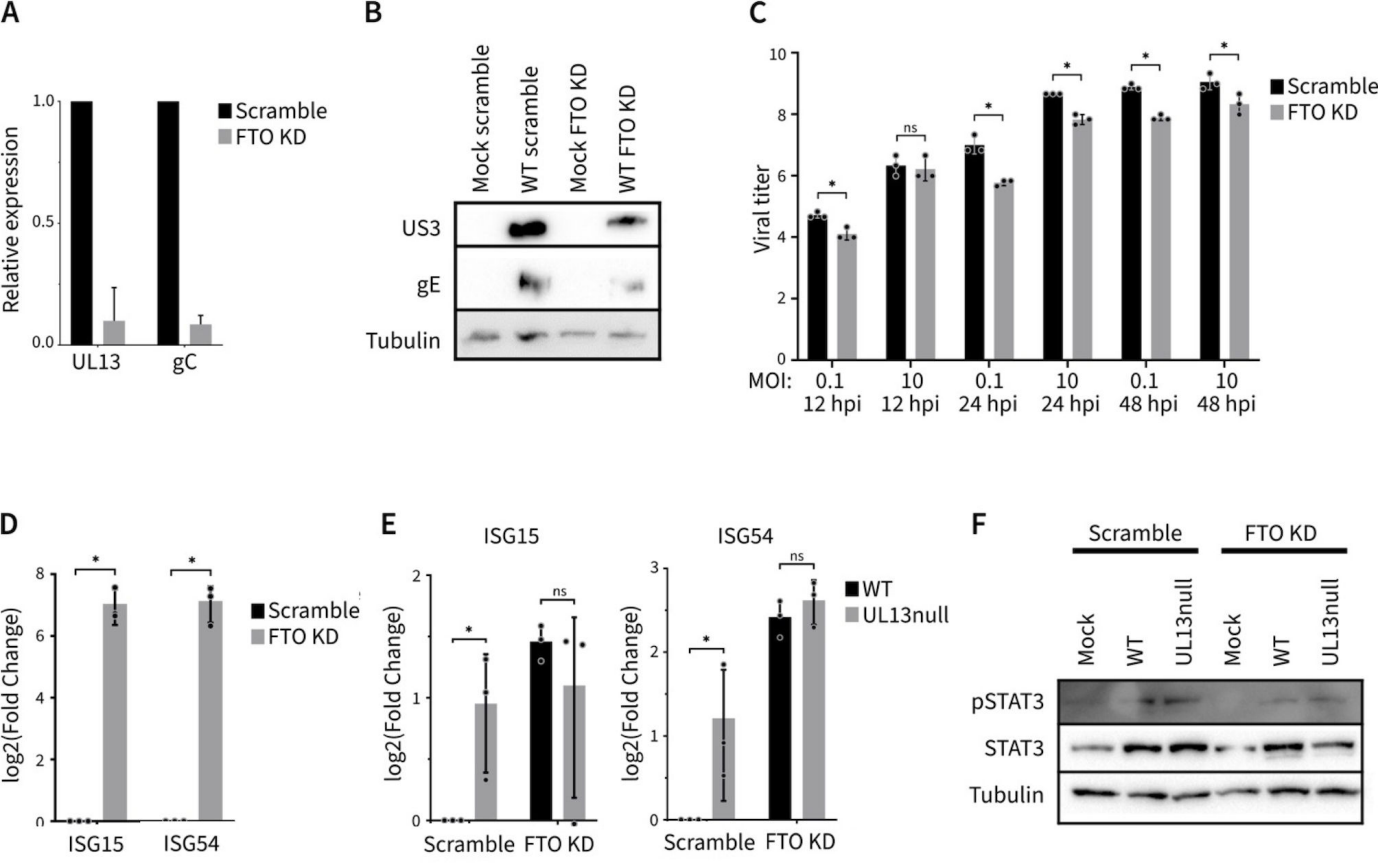
FTO depletion leads to reduced PRV replication, and the UL13 kinase of PRV leads to FTO-dependent suppression of antiviral ISG expression in primary cells. (**A**) Viral early (UL13) and late (gC) mRNA expression levels in WT PRV-infected primary porcine kidney cells at 8 hpi upon siRNA-mediated KD of FTO, relative to cells treated with scrambled siRNA. (**B**) Viral early (US3) and late (gE) protein levels in WT PRV-infected PPK cells at 8 hpi upon siRNA-mediated knockdown of FTO or treatment with scrambled siRNA. (**C**) Infectious virus titers at 12, 24, and 48 hpi in PRV-infected PPK cells with an MOI of 0.1 and 10 upon siRNA-mediated KD of FTO or treatment with scrambled siRNA. Titers are expressed as the log10 of the TCID50/mL. *P < 0.05. (**D**) mRNA expression levels of IFN-stimulated gene 15 (ISG15) and IFN-stimulated gene 45 (ISG54) upon siRNA-mediated knockdown of FTO in PPK cells, relative to cells treated with scrambled siRNA. *P < 0.05. (**E**) mRNA expression levels of ISG15 and ISG54 upon infection with WT or UL13null PRV in PPK cells upon siRNA-mediated knockdown of FTO or treatment with scrambled siRNA, relative to WT-infected scrambled siRNA-treated cells. Cells were treated with the proteasome inhibitor MG132 and IFN-alpha at 2 hpi. *P < 0.05. (**F**) STAT3 and phosphorylated STAT3 (pSTAT3) protein expression in non-infected, WT, or UL13null PRV-infected PPK cells at 8 hpi, upon siRNA-mediated knockdown of FTO or treatment with scrambled siRNA. Cells were treated with the proteasome inhibitor MG132 and IFN-alpha at 2 hpi.

Hence, we examined if FTO knockdown affects PRV mRNA and protein production in IFN-competent primary porcine kidney epithelial cells. In PPK cells, depletion of FTO resulted in a prominent reduction of viral RNA and protein production (Fig. 5A and B). Titration of progeny virus produced in PRV-infected PPK cells showed that knockdown for FTO by siRNA resulted in reduced virus titers compared to PPK cells treated with scrambled siRNA (Fig. 5C).

To investigate whether the impact of FTO knockdown on PRV protein production and PRV titers in PPK cells correlates with differences in ISG expression, we assessed whether siRNA-mediated knockdown of FTO in PPK cells affects ISG expression. Specifically, we analyzed the expression of ISG15 and ISG54 using RT-qPCR. FTO knockdown resulted in increased expression of these transcripts compared to cells treated with control scrambled siRNA, indicating that FTO is involved in the suppression of ISG expression (Fig. 5D). This observation aligns with a recent report that showed a suppressive effect of FTO on ISG expression (54).

We next wished to investigate whether UL13 affects antiviral ISG expression upon type I IFN addition to PRV-infected cells and, if yes, whether this is FTO dependent. Alphaherpesviruses like PRV display a multitude of mechanisms to suppress the antiviral signaling of type I IFN (55, 56). We reported earlier that PRV suppresses type I IFN signaling by degrading Janus kinases in a proteasome-dependent manner and that treatment of infected cells with MG132 is required to yield detectable ISG mRNA levels in PRV-infected cells (37). PPK cells were, therefore, treated with the proteasome inhibitor MG132 and IFN-alpha to allow detectable expression of ISG transcripts during PRV infection. To assess a potential contribution of FTO to the observed effects, cells were treated with either scrambled siRNA or FTO-specific siRNA. In PPK cells treated with scrambled siRNA, we observed higher expression of ISGs in cells infected with UL13null PRV compared to cells infected with WT PRV, indicating that UL13 expression suppresses ISG expression. Upon siRNA-mediated knockdown of FTO, ISG expression levels increased in WT PRV-infected PPK cells and reached levels that were similar as those observed in UL13null PRV-infected cells (Fig. 5E). These results thus reveal that UL13 expression reduces ISG expression in PRV-infected PPK cells and that this depends on FTO, indicating that the impact of UL13 on FTO suppresses the expression of antiviral ISG transcripts.

McFadden et al. (54) showed that FTO negatively regulates STAT3-mediated signaling, which may affect ISG expression (54). Therefore, we hypothesized that the UL13-induced FTO phosphorylation might lead to decreased STAT3 activation as a means to suppress ISG expression. To assess this, we analyzed phosphorylation of STAT3 at Y705. However, we were unable to confirm increased expression of phosphorylated STAT3 upon FTO knockdown (Fig. 5F). A possible explanation for the differences between the current study and the report of McFadden et al. (54) could be the difference in cell types used in each study. Hence, differences in STAT3 activation do not appear to be involved in UL13- and FTO-dependent suppression of ISG expression in PRV-infected cells.

Overall, these data indicate that FTO plays an important role in suppressing the expression of antiviral ISGs and that the UL13 kinase of PRV leads to FTO-dependent suppression of antiviral ISG expression.

### PRV infection leads to UL13-independent suppression of m6Am levels in host snRNA

Next, we wanted to investigate whether the UL13-dependent impact of PRV infection on FTO phosphorylation correlates with possible changes in m6Am levels in RNA. Previous studies have primarily focused on the effect of FTO depletion on m6A and m6Am levels in mRNA in cell culture; however, depletion of FTO appears to elicit only moderate effects in this type of RNA (13, 57). This could be explained by the possibility that the primary target of FTO is not cytosolic mRNA, but rather nuclear RNA, a notion supported by the predominant nuclear localization of FTO. Indeed, Wei et al. (57) and Mauer et al. (6) demonstrated that knockout of FTO results in a significant increase in m6Am-methylated snRNA (6, 57). Therefore, we investigated whether UL13-induced FTO phosphorylation correlates with differences in m6Am levels of host snRNA.

**Fig 6:**
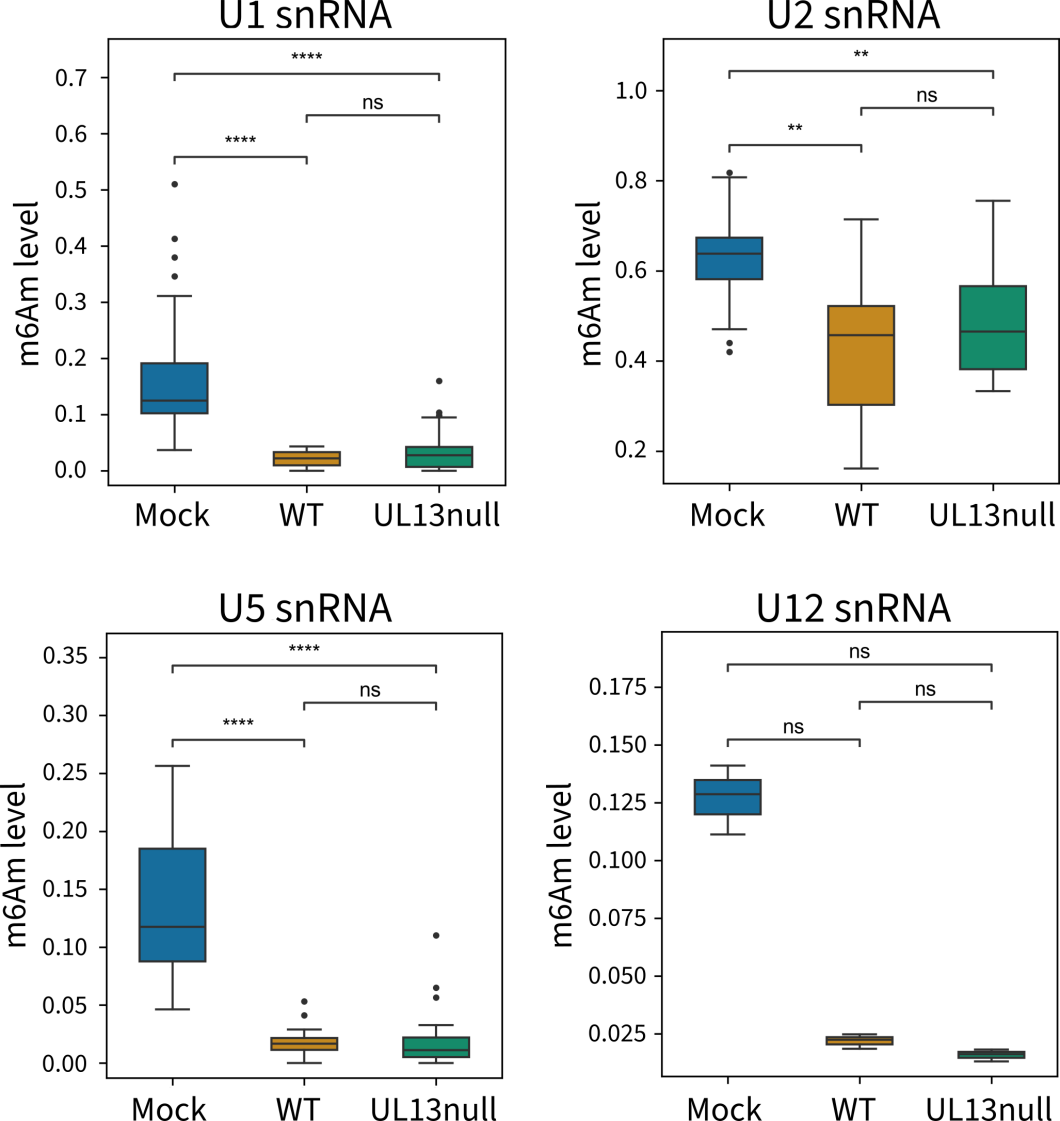
PRV infection leads to a significant UL13-independent reduction of m6Am levels in snRNA. Quantification of m6Am levels in snRNA of mock-infected, wild-type, and UL13null PRV-infected ST cells at 16 hours post-infection, quantified per type of snRNA (U1, U2, U5, and U12 snRNA) (*n* = 3). **P < 0.01 and ****P < 0.0001.

For this purpose, a recently developed technique called CROWN-seq was used (39). CROWN-seq is a quantitative 5′ m6Am mapping method based on the m6A quantifying technique GLORI-seq (40). We determined m6Am levels in snRNA (U1, U2, U5, and U12 snRNA) from mock-infected cells and cells infected with WT or UL13null PRV. We observed a pronounced decrease in m6Am levels in all snRNA in WT PRV-infected cells compared with mock-infected cells. Cells infected with UL13null PRV also show this marked reduction in m6Am levels (Fig. 6). Therefore, PRV infection results in a UL13-independent reduction in m6Am levels in snRNA, suggesting that the UL13- and FTO-mediated impact on ISG expression is unrelated to differences in m6Am methylation in host snRNA.

### PRV triggers phosphorylation of the m6Am methyltransferase PCIF1 in a UL13-independent manner

**Fig 7:**
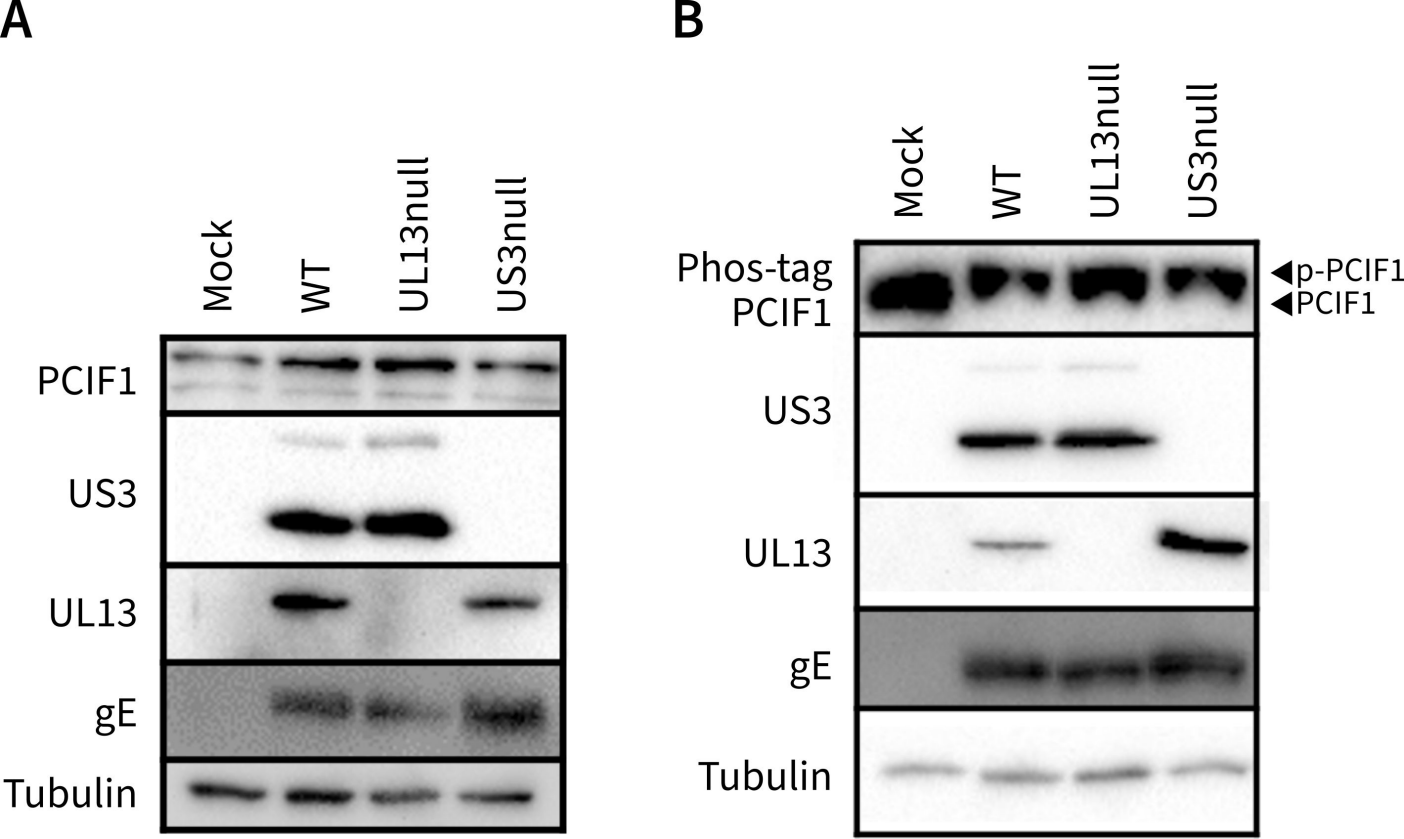
PRV infection triggers phosphorylation of the m6Am writer PCIF1. (**A**) Western blotting of PCIF1 in ST cells mock infected, infected with WT PRV, UL13null PRV, or US3null PRV at 16 hours post-infection. (**B**) Phos-tag assay of PCIF1 in ST cells mock infected, infected with WT PRV, UL13null PRV, or US3null PRV at 16 hpi.

Considering that both the m6Am PCIF1 writer and FTO eraser proteins affect m6Am levels (5, 9), we assessed whether PRV infection has an impact on protein expression of the m6Am methyltransferase PCIF1. Western blot analysis of ST cells infected with PRV revealed no discernible differences in PCIF1 signal (Fig. 7A), indicating that PRV infection does not affect expression levels of the m6Am writer protein.

To explore whether PCIF1 undergoes phosphorylation upon PRV infection, Phos-tag gel assays were performed. This analysis revealed that PRV infection triggers phosphorylation of PCIF1. Infection with UL13null or US3null PRV yielded similar PCIF1 phosphorylation compared to WT infection (Fig. 7B), indicating that phosphorylation of PCIF1 during PRV infection is not caused by either of the two viral protein kinases. Although it remains to be determined if phosphorylation of PCIF1 affects its methylase activity, these data indicate that PRV infection affects the cellular m6Am machinery in several ways.

## DISCUSSION

The discovery of FTO as m6A demethylase was the trigger for the renewed interest in the field of RNA methylation (13), which had been silent for several decades. The potential for trigger-dependent methylation and demethylation of m6A has put RNA methylation at the same level as the epigenome and led to the initiation of the field of epitranscriptomics. It was later realized that m6Am, rather than m6A, may represent the preferential physiological substrate of FTO (5, 14). Another RNA demethylase, ALKBH5, was later shown to specifically demethylate m6A (15). Even though trigger-dependent demethylation of mRNA is still controversial, m6A and m6Am demethylases have been shown to be important in diverse biological contexts, including fertility and dopaminergic signaling, respectively (15, 58). The effect of m6A(m) erasers on virus replication has been investigated for some viruses, including hepatitis B virus , hepatitis C virus, and human immunodeficiency virus (HIV) (59–61). These studies indicate that FTO may exert either pro- or antiviral effects, depending on the virus, but the mechanisms by which it affects virus replication are largely unknown. Although recent work shows that infection with the porcine reproductive and respiratory syndrome virus leads to altered protein expression of m6A(m) eraser proteins and that this correlates with increased m6A levels (62), so far, little research has gone toward possible strategies employed by viruses to influence FTO and/or RNA demethylation.

In this study, we show that the m6A(m) eraser FTO is phosphorylated during PRV infection. Our findings also reveal that, both in porcine and human cells, the anti-FTO antibody ab92821 (Abcam) preferentially recognizes a phosphorylated form of FTO that is more abundantly present during PRV infection. We further show that this phosphorylation of FTO depends on the kinase activity of the viral serine/threonine protein kinase UL13. Expression of this protein kinase, in the absence of virus infection, was sufficient to induce phosphorylation of FTO. Although UL13 homologs are present in all members of the *Orthoherpesviridae* family, the amino acid sequences of these UL13 homologs show only limited similarity (47), and it remains to be identified whether the phosphorylation of FTO is a conserved phenotype in (alpha)herpesvirus infection.

In this study, we found FTO to be strictly nuclear-localized and did not detect any translocation of FTO from the nucleus toward the cytoplasm upon PRV infection. This observation aligns with prior immunofluorescence studies, which have consistently depicted a weak or negligible FTO signal in the cytoplasm (13, 48), even upon overexpression of GFP-tagged FTO (63). Although some studies have proposed the ability of FTO to undergo nucleo-cytoplasmic shuttling (51, 63, 64), the results of others and ourselves do not support this idea (13, 48, 50). Moreover, Jia et al. (13) and Berulava et al. (48) have highlighted the localization of FTO within nuclear speckles and showed colocalization of FTO with several splicing or splicing-related speckle factors (13, 48). These splicing speckles are major nuclear domains enriched in components of the splicing machinery and function in spliceosome assembly or post-transcriptional pre-mRNA splicing (65). This colocalization supports the idea that m6Am in snRNA is a major target of FTO (5, 6, 14). We found that PRV infection results in a very substantial decrease in m6Am levels in host snRNA, which is independent of UL13. It may be interesting to investigate in future research whether the UL13-independent decrease in m6Am levels in host snRNA in PRV-infected cells may be linked to the UL13-independent relocation of FTO to a more punctate intranuclear pattern in PRV-infected cells (Fig. 3). M6Am methylation levels of snRNA have been linked to splicing efficiency and specificity (66). Alphaherpesvirus infection has been reported to disrupt normal host splicing processes (67), and the HSV-1 protein ICP27 modifies pre-mRNA processing of several cellular genes (68–71) by its interaction with U1- and U2-associated proteins (69, 72, 73). Also, HSV-1 infection induces the expression of a spliced variant of the ISG MxA, which was found to support HSV-1 replication rather than inhibit it (74). Hence, it may be worth in future research to analyze the potential impact of PRV-induced suppression of m6Am in snRNA on host cell splicing efficiency and specificity.

Our findings provide additional evidence that the m6A(m) demethylase FTO is an important regulator of the type I IFN response, in line with recent data (54). We show that depletion of FTO in primary cells induces a marked increase in the expression of ISGs, correlating with impaired viral protein production. In addition, we show that the suppressive effect of UL13 on ISG expression in PRV-infected cells depends on FTO, suggesting that PRV-induced and UL13-mediated phosphorylation of FTO constitutes a viral strategy to impair the host antiviral response. Exactly how (phosphorylation of) FTO regulates antiviral IFN/ISG expression is unclear and will require additional research. Since the effect of PRV infection on m6Am levels in snRNA was independent of UL13, this effect may be independent of FTO’s demethylase activity. However, since we specifically analyzed m6Am levels in snRNA, we cannot formally rule out a possible contribution of the demethylase activity of FTO on mRNA in the observed impact of UL13-FTO on ISG expression.

Regardless of the underlying mechanism, our data and those of others indicate a central role for FTO in suppressing ISG expression, which may have an impact beyond virus biology. For example, since recent studies link FTO with particular auto-immune diseases (75), it may be worthwhile to investigate whether potential single nucleotide polymorphisms in FTO that may affect its activity could be associated with IFN-related autoimmune diseases (76). Although speculative, since FTO phosphorylation results in the downregulation of ISG expression at the mRNA level, one possibility may be that FTO possibly acts as a regulatory protein to ensure an adequate type I interferon response. Phosphorylation could inhibit its function, thereby leading to the increased transcription of ISGs, as observed in this study. FTO might, therefore, be involved in regulating proteins within the canonical JAK-STAT pathway, as has been suggested before (54), or potentially in a non-canonical pathway that leads to ISG expression upon virus infection.

Future research will need to reveal which site(s) in FTO are phosphorylated upon UL13 expression and whether UL13 directly phosphorylates FTO or, alternatively, triggers FTO phosphorylation by activating a host kinase. Several phosphorylated amino acids have been identified in FTO (46, 77), and numerous putative phosphorylation sites were suggested by phosphoproteome studies (78). Faulds et al. (46) showed that phosphorylation of FTO at S249 and S253 by the glycogen synthase kinase-3 leads to proteasomal degradation of FTO (46), whereas Hirayama et al. (77) suggested that phosphorylation of FTO at T150 is linked to the export of FTO from the nucleus to the cytoplasm (77). These functions are likely unrelated to the phosphorylation we observed since neither degradation nor translocation of FTO were observed in our assays. Furthermore, the cellular protein kinase C-beta was shown to phosphorylate FTO, linked to suppressed ubiquitin-proteasome degradation of FTO; however, the phosphorylation site of this cellular kinase remains unidentified (79).

In addition to uncovering UL13-dependent phosphorylation of the m6Am eraser FTO, our study shows that PRV infection also triggers phosphorylation of the m6Am writer PCIF1. Despite several high-throughput phosphoproteome studies suggesting phosphorylation of various residues of PCIF1, direct evidence of PCIF1 phosphorylation has been lacking until now (80). It is currently unclear if and how such virus-induced PCIF1 phosphorylation may affect its activity. Since PCIF1 phosphorylation is independent of UL13 and since we observed that the PRV-dependent decrease in m6Am levels in snRNA is independent of UL13, one speculative possibility could be that phosphorylation of PCIF1 upon PRV infection may hamper its activity. Interestingly, recent research by Zhang et al. (81) indicates that HIV infection leads to enhanced degradation of PCIF1, implying potential benefits for viruses to remove or inhibit PCIF1. Future research is aimed at further clarifying this.

In summary, this study unveils that PRV infection leads to phosphorylation of the m6A(m) demethylase FTO, which is triggered by the viral UL13 protein kinase. We demonstrate that FTO is a critical suppressor of antiviral ISG expression and that UL13 expression leads to FTO-dependent inhibition of antiviral ISG expression, pointing to virus-induced phosphorylation of FTO as a potential novel viral IFN evasion strategy. These findings, therefore, add novel insights to our understanding of alphaherpesvirus biology and shed light on (herpes)viral and possibly broader regulatory mechanisms of the type I IFN response and governing m6A(m) methylation.
